# Targeted Krüppel-Like Factor 4 Gene Knock-Out in Retinal Ganglion Cells Improves Visual Function in Multiple Sclerosis Mouse Model

**DOI:** 10.1523/ENEURO.0320-19.2020

**Published:** 2020-03-19

**Authors:** Venu Talla, Rajeshwari Koilkonda

**Affiliations:** Bascom Palmer Eye Institute, Department of Ophthalmology, University of Miami, Miller School of Medicine, Miami, FL 33136

**Keywords:** axon regeneration, demyelination, EAE, MS, neuroprotection, optic neuritis

## Abstract

Axonal demyelination injury and neuronal degeneration are the primary causes of visual disability in multiple sclerosis (MS)-linked optic neuritis patients. Immunomodulatory therapies targeting inflammation have failed to avert the disease progression and no therapies exist to prevent the neuronal deficits seen in MS to date. Neuroprotective strategies targeting oligodendrocytes and astroglia have shown limited success due to a lack of axonal regeneration from injured neurons. In this study, we used the chronic experimental autoimmune encephalomyelitis (EAE) mouse model of MS to investigate the axonal regenerative approach to improve the neuronal function. Our approach focused on targeted knock-out (KO) of the developmentally regulated axon growth inhibitory Krüppel-like factor 4 (*Klf4*) gene in retinal ganglion cells (RGCs) of *Klf4^fl/fl^*mice by intravitreal delivery of AAV2-Cre-ires-EGFP recombinant virus (1) at the time of EAE sensitization and (2) after the onset of optic neuritis-mediated visual defects in the mice. *Klf4* gene KO performed simultaneous with EAE sensitization prevented the visual loss as assessed by pattern electroretinograms (PERGs) in the mice and protected the RGCs from EAE-mediated death. More importantly, however, *Klf4* gene KO after the onset of optic neuritis also resulted in RGC neuroprotection with additional restoration of their function, thereby improving the visual function outcomes in the EAE model. This study establishes the efficacy of *Klf4* targeted knock-down in EAE even after the onset of disease symptoms, and thus should be further explored as a potential treatment strategy for MS/optic neuritis patients.

## Significance Statement

Multiple sclerosis (MS) is a leading cause of permanent disability in young adults in which effective treatment has remained elusive. The neuroprotective strategies targeting oligodendrocytes and astroglia have shown limited success due to a lack of axonal regeneration from injured neurons. In the current study, we explored the axon regenerative approach to treat MS in mouse model and showed that targeted knock-out (KO) of axon growth inhibitory Krüppel-like factor 4 (*Klf4*) gene in RGCs of experimental autoimmune encephalomyelitis (EAE) mice is efficient in preventing not only the EAE-mediated RGC death but also restoring the function of injured axons by facilitating regeneration, and thereby improving the visual function in the mice even after the onset of EAE-mediated visual defects.

## Introduction

Multiple sclerosis (MS) is a chronic inflammatory and neurodegenerative disease of the central nervous system affecting over 0.7–0.9 million people with an estimated 10-year cumulative prevalence of 309.2 per 100,000 adults in the United States (Adelman et al., 2013; Wallin et al., 2019). Experimental autoimmune encephalomyelitis (EAE), induced on administration of central nervous system (CNS) myelin components is one of the most widely used animal models for human MS (Guy and Rao, 1984; Kornek et al., 2000; Owens, 2003). MS/EAE is an inflammatory autoimmune disease characterized by inflammation, demyelination and progressive axonal loss in the CNS, which ultimately leads to permanent disability (Bjartmar and Trapp, 2001). Optic neuritis is one of the most common clinical manifestations of MS and is diagnosed as the initial symptom in ∼25% of MS patients, while 70% develop the condition during the course of the disease (Talman et al., 2010; Balcer et al., 2015). Since neurologic deficits of retinal ganglion cell (RGC)/optic nerve (ON) injury are detected early during the onset of the disease and are more clinically apparent than elsewhere in the CNS, optic neuritis is instrumental for testing neuroprotective approaches for MS therapies and has been used in multiple clinical trials (Aktas et al., 2016).

To date, no effective therapy exists to prevent neuronal deficits associated with MS/optic neuritis, except for recent reports claiming prevention of neurologic deficits in an MS mouse model on intranasal delivery of the biological secretome of amnion-derived multipotent progenitor cells (Khan et al., 2017, 2019; Grinblat et al., 2018). Most of the initial MS research is focused on immunomodulatory therapies targeting the inflammatory and autoimmune components (Noseworthy et al., 2001). However, immunomodulatory drugs have thus far failed to prevent permanent disability in progressive MS due to a lack of axonal regeneration (Polman et al., 2006; Shirani et al., 2012). More recent efforts targeted the early onset axonal and neuronal degenerations, which are linked to disease progression in MS patients and are currently recognized as the primary cause of visual and neurologic disability in MS and optic neuritis patients (Trapp et al., 1998; Lovas et al., 2000; Kuhlmann et al., 2002; Bjartmar et al., 2003; Kutzelnigg et al., 2005). Consequently, research findings on axonal protection/regeneration in MS/EAE helped to re-focus therapeutic strategies to protect axons and their functions, particularly via enhanced regeneration (Bjartmar and Trapp, 2001; Bjartmar et al., 2003).

In contrast to embryonic or neonatal neurons, adult CNS neurons cannot regenerate injured axons (Chen et al., 1995; Moore et al., 2009) possibly due to the inhibitory environment created by mature oligodendrocytes and astroglia (Yiu and He, 2006), which form a glial scar after myelin sheath degeneration in MS/EAE (Steinman et al., 2002; Frohman, 2003). Yet strategies targeting the astroglial inhibitory cues in EAE animal models resulted in only modest regeneration (Bartsch et al., 1995; Kim et al., 2003). However, embryonic neurons are found to readily grow past these inhibitory cues in CNS when transplanted, thus indicating a role for intrinsic developmentally regulated factors in inhibiting the axon growth and regeneration in adult CNS neurons (Lu et al., 2012). Thus, the hypothesis for this study was that the knock-out (KO) of intrinsic axon growth inhibitory factors in injured CNS neurons can promote axon regeneration thereby aiding in the restoration of visual function in MS and optic neuritis. cAMP (Cai et al., 2001), cAMP response element-binding protein (CREB; Gao et al., 2004), B cell lymphoma/leukemia (Bcl-2; Cho et al., 2005), anaphase promoting complex (APC) signaling pathways (Konishi et al., 2004), phosphatase and tensin homology (PTEN; Park et al., 2008), and zinc finger containing Krüppel-like transcription factors (KLFs; Moore et al., 2009) were identified among the most potent intracellular signaling molecules. Among these factors, KLF4 expression was found to be developmentally regulated and was demonstrated to possess a strong inhibitory effect on axonal growth and/or regeneration in injured and healthy adult CNS neurons (Moore et al., 2009). Conditional KO of the *Klf4* gene in rat primary RGCs *in vitro* and in a rat ON crush model *in vivo* resulted in robust axonal regeneration and growth (Moore et al., 2009). For this reason, in the current study we decided to test whether targeted *Klf4* gene deletion after the disease onset could alleviate optic neuritis in EAE-challenged mice.

## Materials and Methods

### Animals

Wild-type (WT) C57BL/6 mice were purchased from The Jackson Laboratory. Initial batch of *Klf4^fl/fl^* mice were a kind gift from Jeffrey L. Goldberg, department of Ophthalmology, Byers Eye Institute at Stanford University, Stanford, CA. Heterozygous B6.129S6-Klf4tm1Khk/Mmmh mice were obtained from MMRRC-MO and were crossed with C57BL/6 to generate homozygous *Klf4^fl/fl^* in C57BL/6 background. Age matched littermate controls were used for each experiment. Animals used were of either sex and number of eyes analyzed for each experiment were shown in Table 1. The protocol for the study was approved by the University Institutional Animal Care and Use Committee and all the procedures were conducted in accordance with the United States Public Health Service Policy on Humane Care and Use of Laboratory Animals.

**Table 1:** Number of eyes analyzed for each experiment

Experiment	Animal	Number of eyes (*N*)
		
Klf4 expression analysis: Western blotting	Mice	18
Cre-GFP immunofluorescence	Mice	3
Electrophysiology (PERG)	Mice	48
Electron microscopy axon counts	Mice	9
RGC numbers immunofluorescence	Mice	9
GAP43 Western	Mice	18

### Plasmid constructs and recombinant AAV2 packaging

SsAAV-CMV-Cre-ires-EGFP is a single stranded AAV plasmid in which the cDNAs of Cre recombinase and eGFP were cloned under cytomegalovirus (CMV) promoter. Presence of internal ribosomal entry site in between cre recombinase and reporter eGFP cDNAs allows, translation of separate Cre recombinase and EGFP proteins from single mRNA transcribed from CMV promotor. Expression of EGFP can be easily monitored with green fluorescence. ScAAV-CMV-mCherry was used as injection control.

Recombinant AAV2 particles carrying ssAAV-CMV-Cre-ires-EGFP or a control scAAV-CMV-mCherry was produced by the plasmid cotransfection method (Hauswirth et al., 2000). Plasmids were amplified and purified using EndoFree plasmid mega kit (QIAGEN Inc.) and were packaged into AAV2 virions with triple tyrosine (Y) to phenylalanine (F) mutations at positions 444, 500, and 730 (Y444F+Y500F+Y730F) in the VP3 capsid by transfection into human HEK 293 cells with standard procedures (Petrs-Silva et al., 2009, 2011). The crude iodixanol fractions were purified using the Pharmacia AKTA FPLC system (GE Healthcare Pharmacia), the virus was then eluted from the column with 215 mM NaCl (pH 8.0), and the recombinant AAV (rAAV) peak was collected. The rAAV-containing fractions were then concentrated and buffer-exchanged in Alcon balanced salt solution with 0.014% Tween 20 (Alcon Laboratories), with a Biomax 100-K concentrator (Millipore). Virus was then titered for DNase-resistant viral genomes by real-time PCR relative to a standard. Finally, the purity of the virus was validated by silver-stained SDS/PAGE, assayed for sterility and lack of endotoxin and then aliquoted and stored at −80°C (Hauswirth et al., 2000).

### Induction of EAE and intravitreal injections

Double floxed *Klf4* adult mice and the littermates were sensitized for EAE by injecting 0.1 ml of sonicated homologous spinal cord emulsion in complete Freunds adjuvant (CFA; Difco) subdermally into the nuchal area. Control littermates received subdermal inoculation with CFA. For intraocular injection of rAAV, mice were sedated by inhalation with 1.5–2% isoflurane. A local anesthetic (proparacaine hydrochloride) was applied topically to the cornea and the pupils were dilated by using a drop of 1% tropicamide. A 32-Gauge needle attached to a Hamilton syringe was inserted through the pars plana under the dissecting microscope. *Klf4^fl/fl^* mice sensitized for EAE received bilateral intravitreal injections of 1 μl ssAAV-CMV-Cre-ires-EGFP (4.95 × 10^12^ vg/ml). EAE-sensitized littermates received 1 μl of scAAV-CMV-mCherry (9.41 × 10^11^ vg/ml) as disease controls. CFA animals received 1 μl of scAAV-CMV-mCherry (9.41 × 10^11^ vg/ml) as injection controls and/or age matched unsensitized mice served as additional controls. For clarity, we now on use following terminology for describing these three groups as (1) EAE-*Klf4^fl/fl^*-Cre-ttd, (2) EAE-vehicle (Veh)-ttd, and (3) control-Veh-ttd mice groups.

**Figure 1.**
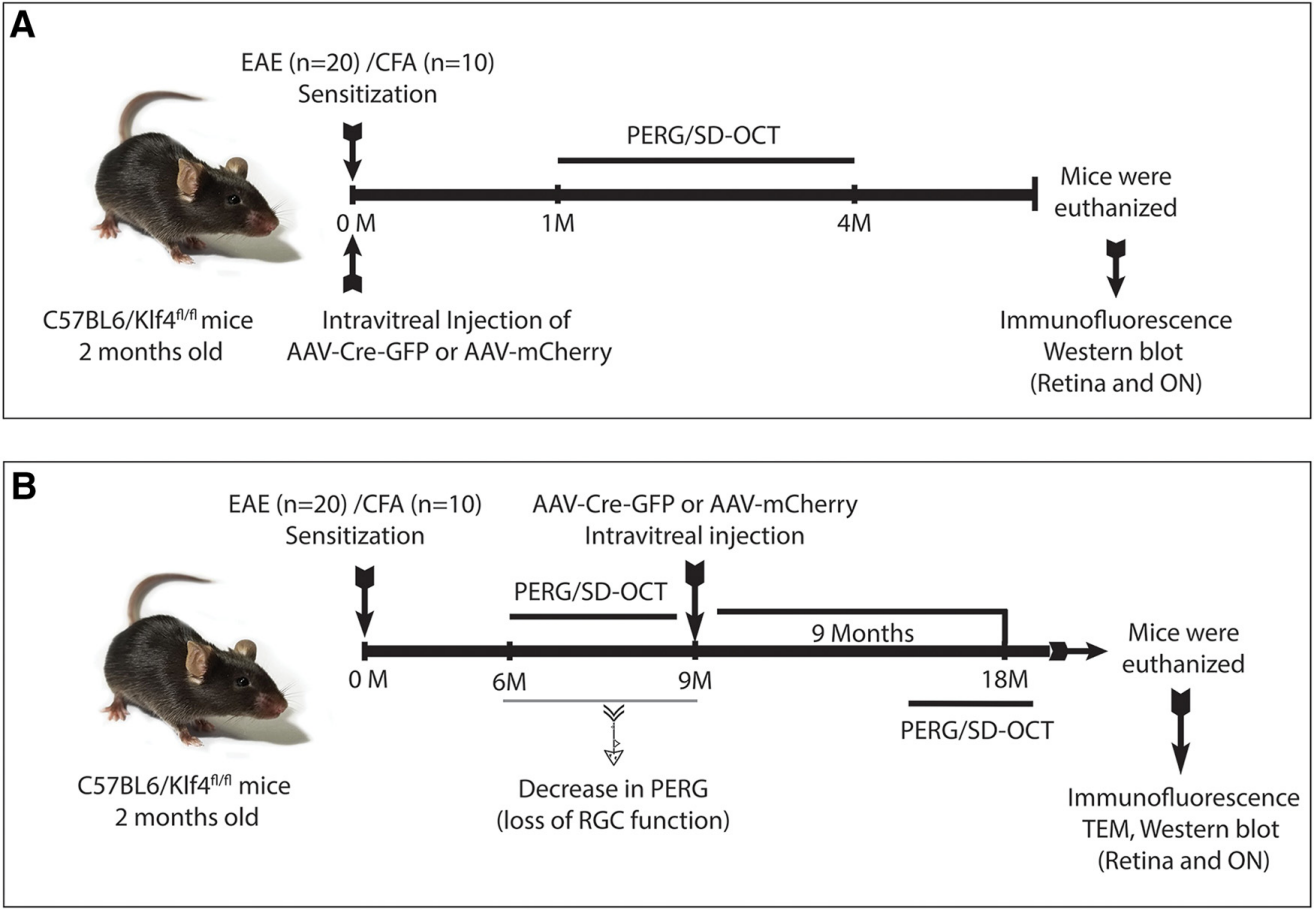
Schematic showing experimental design. ***A***, Experimental design for simultaneous EAE sensitization and rescue injections (Group I). ***B***, Experimental design for Group II mice experiments where rescue injections were performed after the significant decrease in PERGs. PERG, Pattern electroretinograms; SD-OCT, Spectral domain optical coherence tomography.

We have performed two separate sets of experiments as shown in experimental schematic diagram (Fig. 1). In the first set of experiments (Group I), EAE sensitization and intravitreal injections were performed simultaneously (Fig. 1*A*). Whereas, in the second set of experiments (Group II) mice were first sensitized for EAE and were followed up for a significant drop in PERG amplitudes compared with CFA controls and then the intravitreal injections were performed (Fig. 1*B*).

### Pattern electroretinography (PERG)

Serial PERGs were obtained at various time points for all mice groups, control-Veh-ttd (*n* = 16 eyes), EAE-Veh-ttd (*n* = 16 eyes), and EAE-*Klf4^fl/fl^*-Cre-ttd mice (*n* = 16 eyes) as shown in schematic (Fig. 1). PERGs in the mouse was recorded as reported by Porciatti group (Nagaraju et al., 2007; Porciatti, 2007; Porciatti et al., 2007). Briefly, mice were weighed and anesthetized by intraperitoneal injection of ketamine/xylazine hydrochloride solution (ketamine 80 mg/kg body weight and xylazine 10 mg/kg body weight). Mice were gently restrained with the use of bite bar and a nose holder to allow unobstructed vision. Body temperature was maintained at 37°C using a feedback-controlled heating pad. The eyes of anesthetized mice were wide open and steady, with un-dilated pupils pointing laterally and upward. The ERG electrode (0.25-mm diameter silver wire configured to a semicircular loop of 2-mm radius) was placed on the corneal surface and it was positioned in such a way as to encircle the pupil without limiting the field of view. Reference and ground electrodes made of stainless-steel needles were inserted under the skin of the scalp and tail, respectively. A small drop of balanced saline was topically applied on the cornea to prevent drying during recording. A visual stimulus of contrast-reversing bars (field area, 50° × 58°; mean luminance, 50 cd/m^2^; spatial frequency, 0.05 cyc/°; contrast, 100%; temporal frequency, 1 Hz) was aligned with the projection of the pupil at a distance of 20 cm. Retinal signals were amplified by 10,000-fold and bandpass filtered by 1–30 Hz. Three consecutive responses to each of the 600 contrast reversals were recorded and checked for their consistency by superimposition and then were averaged. Averaged PERG values were analyzed to evaluate the major positive and negative waves using commercially available software (Sigma Plot; Systat Software Inc.).

### AAV-mediated Cre recombinase, EGFP expression, and *Klf4* KO and growth-associated protein 43 (GAP43) expression in mice RGCs

#### Western blotting

Total protein fractions isolated from retina and ONs from the control mice and *Klf4^fl/fl^*mice euthanized at one-month postinjection (MPI) of ssAAV-CMV-Cre-ires-EGFP were used for KLF4 protein expression analysis (*n* = 6 eyes/group). Retina and ON protein samples isolated from 9 MPI to 18 MPS of control-Veh-ttd mice (*n* = 6 eyes), EAE-Veh-ttd (*n* = 6 eyes), and EAE-*Klf4^fl/fl^*-cre-ttd (*n* = 6 eyes) mice were used for GAP43 expression analysis. Briefly, tissues were washed twice in ice cold PBS, resuspended in Pierce RIPA buffer (Thermo Scientific) containing Pierce protease inhibitor (Thermo Scientific), homogenized using hand held battery operated tissue homogenizer (VWR International LLC), quantified using Bio-Rad Dc protein assay kit (Bio-Rad) and stored at −20°C until use. Equal amounts of protein were loaded on the 4–12% NuPAGE Bis-Tris gels (Invitrogen) and were electro-transferred onto PVDF membranes. Membranes were blocked in TBST containing 5% non-fat dry milk and 0.5% Tween 20 for 1 h and incubated with respective primary antibodies that included rabbit polyclonal to GFP (Abcam catalog #ab290, RRID:AB_303395), rabbit polyclonal to KLF4 (Abcam catalog #ab72543, RRID:AB_1269261), rabbit polyclonal to GAP43 (Abcam catalog #ab16053, RRID:AB_443303), and rabbit polyclonal to GAPDH (Cell Signaling Technology catalog #2118, RRID:AB_561053). The membranes were washed three times with TBST buffer and incubated with the goat anti rabbit IgG conjugated with horseradish peroxidase (HRP) secondary antibody (Santa Cruz Biotechnology). Membranes were then washed in TBST buffer three times and immunodetected using the enhanced chemiluminescence (ECL) system (GE Healthcare). Band intensities were quantitated by using ImageJ-NIH software.

### Immunofluorescence

One month after intravitreal injection of ssAAV-CMV-Cre-ires-EGFP, *Klf4^fl/fl^*mice were euthanized and eyeballs and ONs were fixed in 4% PFA for 1 h (*n* = 3 eyes/group). Retina and ONs were dissected out and retinal whole mounts and cryosections of retina and ONs were immuno-stained for rabbit polyclonal to GFP (Abcam catalog #ab290, RRID:AB_303395) and rat monoclonal to Thy1 (Abcam catalog #ab85352, RRID:AB_1861402) antibodies and were imaged on Zeiss LSM 700 confocal microscope (Zeiss LSM 700; Zeiss).

### Histology and ultrastructure

Eighteen months after EAE sensitization and nine months post rAAV rescue injections, all the mice were overdosed with sodium pentobarbital. They were then perfused by cardiac puncture with PBS and then with fixative consisting of 4% paraformaldehyde in 0.1 M PBS buffer (pH 7.4).

### RGC counts-immunofluorescence

The eyeballs and ONs were dissected out and fixed in 4% paraformaldehyde in PBS for 1 h and then 0.4% paraformaldehyde overnight (*n* = 3 eyes/group). Cornea and lens were removed, and the retina was dissected from the eyecup. Four radial cuts were made to flatten the retina. After three washes in PBS, the retinal tissues were permeabilized with 0.5% Triton X-100 (Dow Chemical Corporation) in PBS for 1 h, then blocked with 10% goat serum containing 0.5% Triton X-100 for 1 h. The flat-mounted retinas were rinsed in PBS and incubated with a rabbit monoclonal anti-Tuj1 (1:200; Covance catalog #MRB-435P-100, RRID:AB_663339) antibody overnight at 4°C. Flat mounted retinas were washed with PBS three times (5 min each) and incubated in 1:500 dilution of goat anti-rabbit Cy3 (Tuj1) or goat anti-rabbit Cy2 antibodies (Jackson ImmunoResearch), along with 4”,6-diamidino-2-phenylindole (2 μg/ml; Santa Cruz Biotechnology Inc.), at 4°C overnight. The working concentrations of primary and secondary antibodies were prepared in 10% goat serum in PBS (pH 7.4) containing 0.2% Triton X-100. The retinas were washed three times (5 min each time) in PBS and then transferred to glass slides with the RGC layer facing up. Retinal tissues were observed under a confocal microscope (Zeiss LSM 700; Zeiss). Images were obtained from all four quadrants of the retina and the number of Tuj1-positive cells were counted and mean ± SE number of cells per mm^2^ were evaluated and plotted as box and whisker plots.

### Ultrastructural analysis

The eyes with attached ONs (*n* = 3 eyes/group) were dissected out and further processed by immersion fixation in 2.5% glutaraldehyde, postfixed in 1% osmium tetroxide, 0.1 M sodium cacodylate-HCl buffer (pH 7.4), 7% sucrose (cold), and then dehydrated through an ethanol series to propylene oxide, infiltrated, and embedded in epoxy resin that was polymerized at 60°C overnight. Semi-thin sections (0.5 μm) of the ONs were obtained and stained with toluidine blue for light microscopic examination. Ultrathin sections (90 nm) were also obtained from all the mice and were placed on nickel grids and imaged on JEOL JEM-1400 transmission electron microscope, operating at 80 kV. Forty images from each ON were imaged randomly covering the entire ON region by an individual masked for the treatment groups. Axon counts, axon diameters, myelin thickness and mitochondrial number and diameters were analyzed using ImageJ-NIH software.

### Statistical analyses

Statistical analyses were performed using GraphPad Prism 6 software. All datasets were analyzed using the KS normality test for normality. One-way ANOVA followed by Tukey’s multiple comparison tests with multiplicity adjusted *p* values were used to compare when more than two groups were present. Two-way ANOVA followed by Tukey’s multiple comparison tests were used to compare the groups when time point is another variable. For axon distribution, myelin thickness and mitochondrial size analyses, we performed two-way ANOVA followed by *t* test, and statistical significance was determined using the Holm–Sidak method, with α = 5%. Computations assume that all rows are samples from populations with the same scatter (SD). All comparison tests were two-tailed; *p* < 0.05 was considered statistically significant. Values were expressed as mean ± SE.

## Results

### SsAAV-CMV-Cre-ires-EGFP efficiently transduces RGCs and KO the KLF4 expression

**Figure 2.**
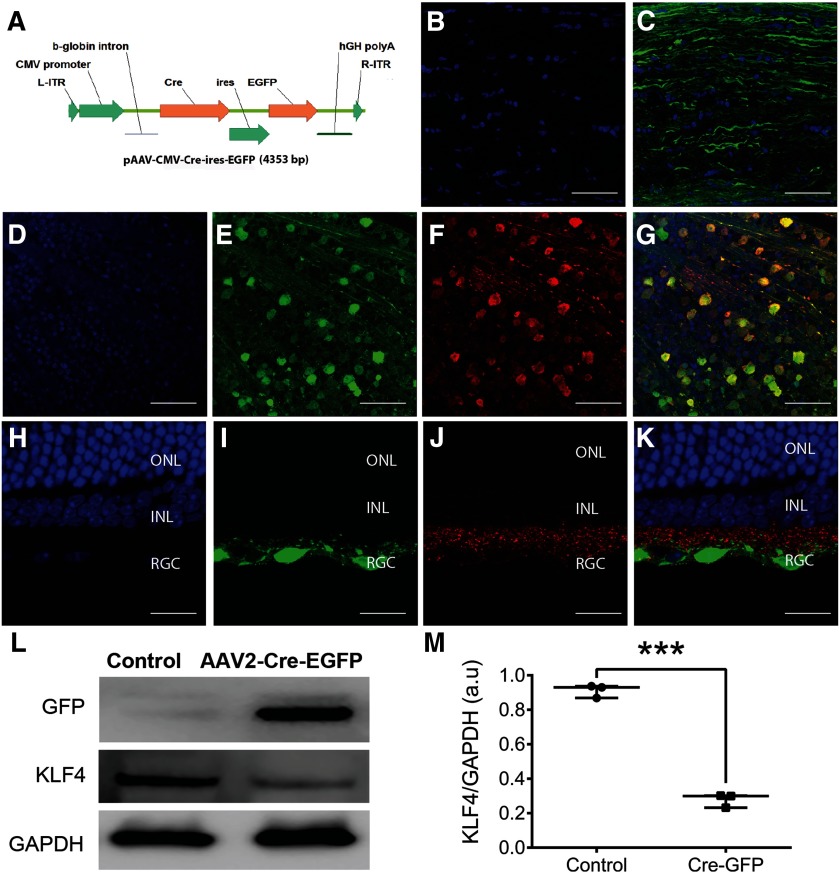
Conditional KO of *Klf4* gene in the retina using rAAV2-Cre-GFP. ***A***, Schematic of the AAV2-Cre-EGFP vector. L-ITR: Left inverted terminal repeat; R-ITR: right inverted terminal repeat; Cre: Cre recombinase; ires: internal ribosome entry site; EGFP: enhanced green fluorescent protein; hGH poly A: bGH Poly adenylation signal. ***B***, ***C***, Immunostaining of ON longitudinal sections of one month after AAV-Cre-ires-EGFP injection mice showed DAPI-labeled ON resident cells (***B***) and GFP-labeled axon bundles (***C***). ***D–G***, Immunostaining of retinal whole mounts at one month after AAV-Cre-ires-EGFP injection showed DAPI-labeled cell nuclei (***D***), GFP-positive cells (***E***), Thy1.2-positive RGCs (***F***), and co-localization of GFP in RGCs shown in merged image (***G***). ***H–K***, Corresponding longitudinal sections showing DAPI-positive nuclei in all three retinal layers (***H***), GFP-expressing cells in RGC layers (***I***), stained positive for Thy1.2 RGC marker (***J***), and all three channels merged (***K***); *n* = 3 eyes. ***L***, Immunoblotting of retina samples obtained one month after intravitreal injection of scAAV-CMV-Cre-ires-EGFP into *Klf4^fl/fl^* mice shows selective expression of GFP and decreased expression of KLF4 compared with control retina. The housekeeping GAPDH expression is shown in injected versus control retina. ***M***, Box-whisker plot shows ImageJ quantitation of KLF4 knock-down in AAV-cre-GFP versus control. Box-plot elements include, center line, median; box limits, upper 75th and lower 25th percentile of the data; whiskers, lowest and highest data points. Samples derived from the same experiment and that gels/blots were processed in parallel for quantitative analysis. ONL, outer nuclear layer; INL, inner nuclear layer; RGC, RGC layer (***p = 0.0001–0.0009; unpaired Student’s *t* test; *n* = 6 eyes/group). *Figure Contributions*: Venu Talla acquired the data and generated the figure.

Immunofluorescence analysis was performed on ON longitudinal sections and retinae obtained one-month postintravitreal injection of ssAAV2-CMV-Cre-ires-EGFP (Fig. 2*A*) in *Klf4^fl/fl^* mice. Longitudinal sections of ON show DAPI-labeled resident cell nuclei (Fig. 2*B*) and abundant expression of GFP in RGC axonal bundles (Fig. 2*C*). Retinal whole mounts of the Cre-GFP-injected mice showed the DAPI-labeled cell nuclei (Fig. 2*D*) expressing GFP (Fig. 2*E*) in Thy1.2-positive RGCs (Fig. 2*F*) and merged image (Fig. 2*G*). Longitudinal sections of the retina show DAPI-positive nuclei in all three retinal layers (Fig. 2*H*). However, GFP expression was limited to the cells in the RGC layer (Fig. 2*I*) that were positive for the Thy 1.2 RGC marker (Fig. 2*J*) and merged image (Fig. 2*K*). Immunoblotting of retina samples showed expression of GFP in the mice injected with ssAAV2-CMV-Cre-ires-EGFP and was absent in un-injected control mice (Fig. 2*L*). Reduced expression of KLF4 was evident in *Klf4^fl/fl^* mice injected with ssAAV2-CMV-Cre-ires-EGFP compared with un-injected control mice while the GAPDH levels remained similar in both the groups (Fig. 2*L*). ImageJ analysis showed a 70% reduction in the KLF4 band intensity in ssAAV-CMV-Cre-ires-EGFP-injected mice compared with uninjected controls (*p* < 0.001; Fig. 2*M*), indicating cre recombinase mediated KO of KLF4 in RGCs and ONs of ssAAV-CMV-Cre-ires-EGFP-injected mice.

### *Klf4* KO in RGCs rescues RGC and on function in EAE mice: PERG

**Figure 3.**
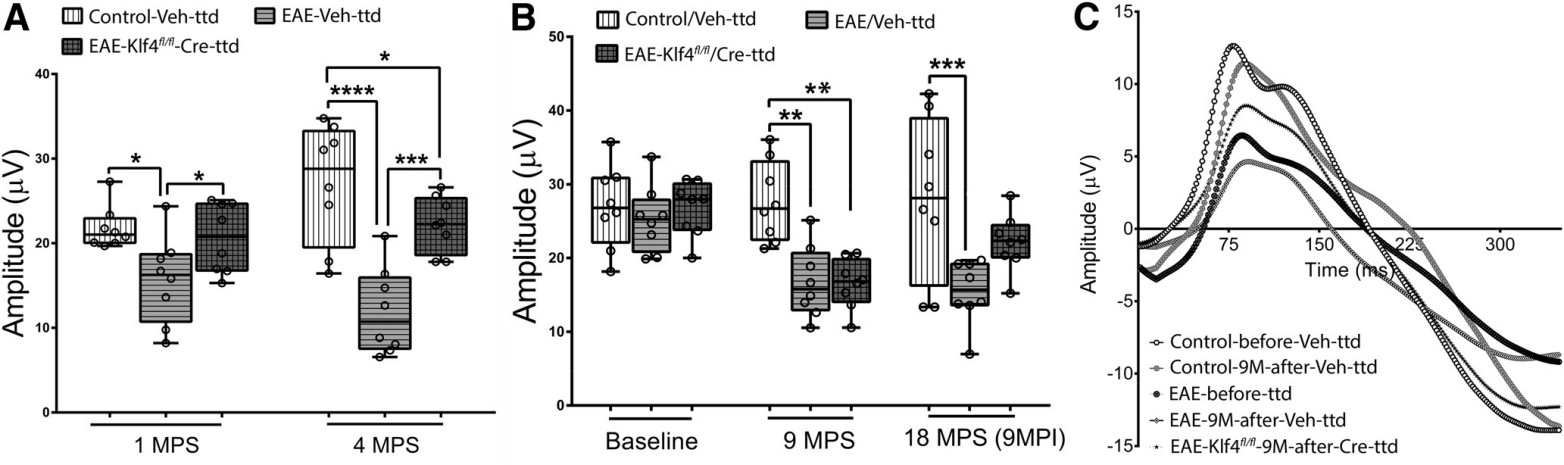
PERG analysis, rescue of visual function. ***A***, Box and whisker plot showing PERG amplitudes of control-Veh-ttd, EAE-Veh-ttd, and EAE-*Klf4^fl/fl^*-Cre-ttd mice at 1 and 4 MPS where EAE sensitization and intravitreal injections were done simultaneously (*n* = 8 eyes/group). ***B***, Box and whisker plot showing PERG amplitudes of Control, EAE and EAE-*Klf4^fl/fl^* mice at 9 MPS versus 18 MPS + 9 MPI of Veh-ttd or Cre-ttd mice. Box-plot elements include, center line, median; box limits, upper 75th and lower 25th percentile of the data; whiskers, lowest and highest data points. ***C***, Representative PERG wave forms of CFA, EAE and EAE *Klf4^fl/fl^* mice at 9 MPS versus 18 MPS + 9 MPI of scAAV-CMV-mCherry or ssAAV-CMV-Cre-ires-GFP. PERGs were recorded three times for each mice/time point and mean PERGs with subtracted background was used for final analysis. Statistical analysis was performed by two-way ANOVA followed by Tukey’s multiple comparison test and *p* values were adjusted for multiple sample comparisons, *p* < 0.05 was considered to be statistically significant; *p = 0.01–0.05; **p = 0.001–0.009; ***p = 0.0001–0.0009, ****p < 0.0001; (*n* = 8 eyes for each group).*Figure Contributions*: Venu Talla acquired the data, analyzed, and generated the figure.

PERG, a sensitive measure of RGC function, was used to evaluate the visual function and progression of optic neuritis in mice. PERGs were recorded for all three groups of mice, i.e., EAE-*Klf4^fl/fl^*-Cre-ttd mice, EAE-Veh-ttd mice and control-Veh-ttd mice at 1 MPI and 4 MPI (Group I; Fig. 3*A*). Two-way ANOVA followed by Tukey’s multiple comparison test with *p* values adjusted for multiple groups of animals over time showed highly significant difference among the control-Veh-ttd, EAE-Veh-ttd, and EAE-*Klf4^fl/fl^*-Cre-ttd mice at one and four months postsensitization (MPS; Table 2). Compared with control-Veh-ttd mice, PERG amplitudes of EAE-Veh-ttd mice decreased progressively from 28%, at one month (*p* = 0.011) to 56% at four months (*p* < 0.0001). On the other hand, Cre recombinase-mediated KO of *Klf4* gene in RGCs of EAE-*Klf4^fl/fl^*-Cre-ttd mice rescued PERG amplitudes significantly compared with EAE-Veh-ttd mice at both the time points tested (*p* = 0.038 at one month and *p* = 0.0001 at four months). On the other hand, PERG amplitudes of EAE-*Klf4^fl/fl^*-Cre-ttd mice at one month were comparable to control-Veh-ttd mice but decreased significantly at four months (*p* = 0.04; Fig. 3*A*; Table 2).

**Table 2:** PERG amplitudes-Group I

Group I (mice groups)	Time points (MPI)	Amplitude (μV; mean ± SEM)	KS normality test (*p* value)	Two-way ANOVA (*p* value)	Tukey’s multiple comparison test *p* value and 95% confidence interval (CI)		
		
					Control-Veh-ttd (vs) EAE-Veh-ttd	Control-Veh-ttd (vs) EAE-*Klf4^fl/fl^*-Cre-ttd	EAE-Veh-ttd (vs) EAE*Klf4^fl/fl^*-Cre-ttd
							
Control-Veh-ttd	1	21.78 ± 0.89	>0.1	Row factor: *p* = 0.53; column factor: ***p* < 0.0003;** interaction: row factor x column factor: ***p* < 0.0098**	***p* = 0.0110** CI: 1.416 to 10.75	*p* **=** 0.796 CI: –3.513 to 5.823	***p* = 0.038** CI: –9.597 to –0.2614
EAE-Veh-ttd	1	15.69 ± 1.83	>0.1				
EAE *Klf4^fl/fl^*- Cre-ttd	1	20.62 ± 1.48	>0.1				
Control-Veh-ttd	4	27.1 ± 2.49	>0.1		***p* < 0.0001** CI: 10.51 to 19.84	***p* = 0.040** CI: 0.2089 to 9.545	***p* = 0.0001** CI: –14.96 to –5.628
EAE-Veh-ttd	4	11.93 ± 1.80	>0.1				
EAE-*Klf4^fl/fl^*- Cre-ttd	4	22.2 ± 1.16	>0.1				

Bold indicates statistically significant difference of *p* values.

We next looked at the PERG amplitudes of the EAE-sensitized *Klf4^fl/fl^* mice that received ssAAV-CMV-Cre-ires-EGFP intravitreal injections following a significant drop in PERG amplitudes (9MPS) and compared with the control and EAE-sensitized littermates mock treated with AAV2 mCherry (Group II mice). At baseline (i.e., before sensitization) the mean PERG amplitudes were indistinguishable among all three groups (i.e., control, EAE, and EAE-*Klf4^fl/fl^* mice; Table 3). However, at 9 MPS, two-way ANOVA indicated significant differences in mean PERG amplitudes among control, EAE littermates and EAE-sensitized *Klf4^fl/fl^* mice (Table 3). While the drop in PERG amplitudes among EAE littermates and EAE *Klf4^fl/fl^* mice were comparable (*p* = 0.99), these two groups showed significantly decreased PERG amplitudes compared with controls at 9MPS (*p* ≤ 0.001; Fig. 3*B*,*C*; Table 3). At this time, EAE-sensitized *Klf4^fl/fl^* mice received intravitreal injection of ssAAV-CMV-Cre-ires-EGFP into both eyes whereas, the EAE littermates and controls received scAAV2-CMV-mCherry injections. Nine months after AAV injection (9 or 18 MPS) PERGs were recorded again for all the groups and amplitudes were compared. At 9 MPI, the EAE-Veh-ttd mice continued to show a significant drop in PERG amplitudes compared with control-Veh-ttd mice (*p* = 0.0002) whereas, the PERG amplitudes were improved in EAE-*Klf4^fl/fl^*-Cre-ttd mice and were comparable to control-Veh-ttd group (*p* = 0.092; Table 3). These PERG results from Group I and Group II mice indicated that Cre-GFP-mediated KO of *Klf4^fl/fl^* gene in EAE/optic neuritis mice not only prevents RGC loss, but also restored the function of remaining, albeit injured RGCs.

**Table 3:** PERG amplitudes-Group II

Group II (mice groups)	Time points	Amplitude(μV; mean ± SEM)	KS normality test (*p* value)	Two-way ANOVA (*p* value)	Tukey’s multiple comparison test *p* value and 95% confidence interval (CI)		
		
					Control/Veh-ttd (vs) EAE/Veh-ttd	Control/Veh-ttd (vs) EAE-*Klf4^fl/fl^*/Cre-ttd	EAE/Veh-ttd (vs) EAE *Klf4^fl/fl^*/ Cre-ttd
							
Control	Baseline	26.93 ± 1.99	>0.1	Row factor: ***p* = 0.0100;** column factor: ***p* = 0.0003;** interaction: row factor x column factor: ***p* = 0.028**	*p* = 0.808 CI: –5.102 to 8.54	*p* = 0.99 CI: –6.624 to 7.022	*p* = 0.846 CI: –8.344 to 5.301
EAE	Baseline	25.21 ± 1.60	>0.1				
EAE-*Klf4^fl/fl^*	Baseline	26.74 ± 1.31	>0.1				
Control	9 MPS	27.60 ± 1.92	>0.1		***p* = 0.001** **CI:** 4.037 to 17.68	***p* = 0.001** **CI:** 4.264 to 17.91	*p* = 0.99 CI: –6.596 to 7.049
EAE	9 MPS	16.74 ± 1.70	>0.1				
EAE-*Klf4^fl/fl^*	9 MPS	16.52 ± 1.19	>0.1				
Control-Veh-ttd	18 MPS (9 MPI)	28.11 ± 3.87	>0.1		***p* = 0.0002** **CI:** 5.819 to 19.46	*p* = 0.092 CI: –0.8202 to 12.82	*p* = 0.057 CI: –13.46 to 0.1831
EAE-Veh-ttd	18 MPS (9 MPI)	15.46 ± 1.52	>0.1				
EAE-*Klf4^fl/fl^* - Cre-ttd	18 MPS (9 MPI)	22.10 ± 1.36	>0.1				

Bold indicates statistically significant difference of *p* values.

### *Klf4* KO in RGCs prevents EAE-mediated RGC loss

**Figure 4.**
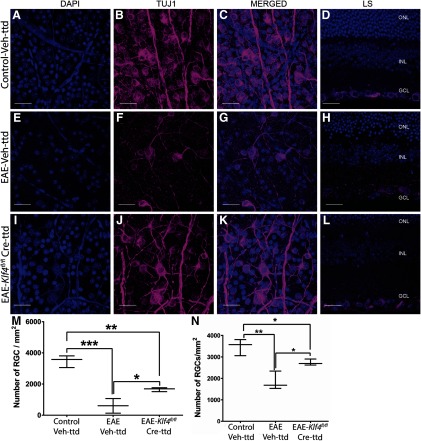
Retinal whole mounts and longitudinal sections: confocal microscopy of retinal whole mounts and corresponding longitudinal sections of (***A–D***) control-Veh-ttd, (***E–H***) EAE-Veh-ttd, and (***I–L***) EAE-*Klf4^fl/fl^*-Cre-ttd mice showing. (***A***, ***E***, ***I***) DAPI-positive cell nuclei, with (***B***, ***F***, ***J***) Tuj1-positive RGCs, along with (***C***, ***G***, ***K***) DAPI and Tuj1 merged images and (***D***, ***H***, ***L***) representative retinal longitudinal sections with DAPI- and Tuj1-positive RGCs. Box and whisker plot (***M***) compare the RGC counts in control-Veh-ttd, EAE-Veh-ttd, and EAE-*Klf4^fl/fl^*-Cre-ttd mice groups, in which ntravitreal injections were done after the PERGs were significantly decreased due to EAE. ***N***, Compares the RGC counts in control-Veh-ttd, EAE-Veh-ttd, and EAE-*Klf4^fl/fl^*-Cre-ttd mice groups, in which intravitreal injections and EAE sensitizations were done simultaneously. Box-plot elements include, center line, median; box limits, upper 75th and lower 25th percentile of the data; whiskers, lowest and highest data points. ONL, outer nuclear layer; INL, inner nuclear layer; RGC, RGC layer. Statistical analysis was performed by one-way ANOVA followed by Tukey’s multiple comparison test, *p* < 0.05 is considered to be statistically significant; *p = 0.01–0.05; **p = 0.001–0.009, ***p = 0.0001–0.0009; *n* = 3 eyes/group. Scale bars: 100 μm.*Figure Contributions*: Venu Talla acquired the images. Rajeshwari Koilkonda did the RGC counting. Venu Talla generated the figure.

The number of RGCs in retinal whole mounts were quantified by immunostaining with Tuj1 antibody. Representative confocal images of retinal whole mounts and corresponding longitudinal sections shows Tuj1-stained RGCs in control-Veh-ttd mice (Fig. 4*A–D*), EAE-Veh-ttd mice (Fig. 4*E–H*) and EAE-*Klf4^fl/fl^*-Cre-ttd mice (Fig. 4*I–L*), at 18 MPS. Quantitative analysis revealed 82% loss of Tuj1-positive RGCs in EAE-Veh-ttd (602 ± 271 per mm^2^, average ± SE, *p* = 0.0002) and 52% loss in EAE-*Klf4^fl/fl^*-Cre-ttd mice (1657 ± 74 per mm^2^; *p* = 0.002) compared with control-Veh-ttd mice (3482 ± 222 per mm^2^; Fig. 4*M*). This correspond to a 37% rescue in RGC numbers in EAE-*Klf4^fl/fl^*-Cre-ttd mice when compared with EAE-Veh-ttd mice (*p* = 0.02; Fig. 4*M*). However, in the case of the first group of experiments where rescue and sensitization were done simultaneously, 48% (*p* = 0.001) of RGCs were lost in EAE-Veh-ttd and 23% (*p* < 0.05) loss in EAE-*Klf4^fl/fl^*-Cre-ttd mice when compared with control-Veh-ttd mice (Fig. 4*N*). This corresponds to a 52% rescue in RGCs of EAE-*Klf4^fl/fl^*-Cre-ttd mice compared with EAE-Veh-ttd mice (*p* < 0.05). In summary, RGC loss was considerably lower in EAE-*Klf4^fl/fl^*-Cre-ttd mice compared with EAE littermates regardless of time at which rescue injection was performed (simultaneously with EAE sensitization vs after PERG amplitude dropped). Higher RGC survival in Group I mice or rescue before injury is as expected. In addition, increased RGC preservation in Cre-GFP injected EAE mice retina corroborates with improved RGC function as observed in our PERG experiments. Next, we checked whether *Klf4* KO in EAE mice RGCs induce axonal regeneration that contributes to the visual function improvement in EAE mice.

### *Klf4* KO in RGCs induces axonal regeneration in EAE mice ONs

**Figure 5.**
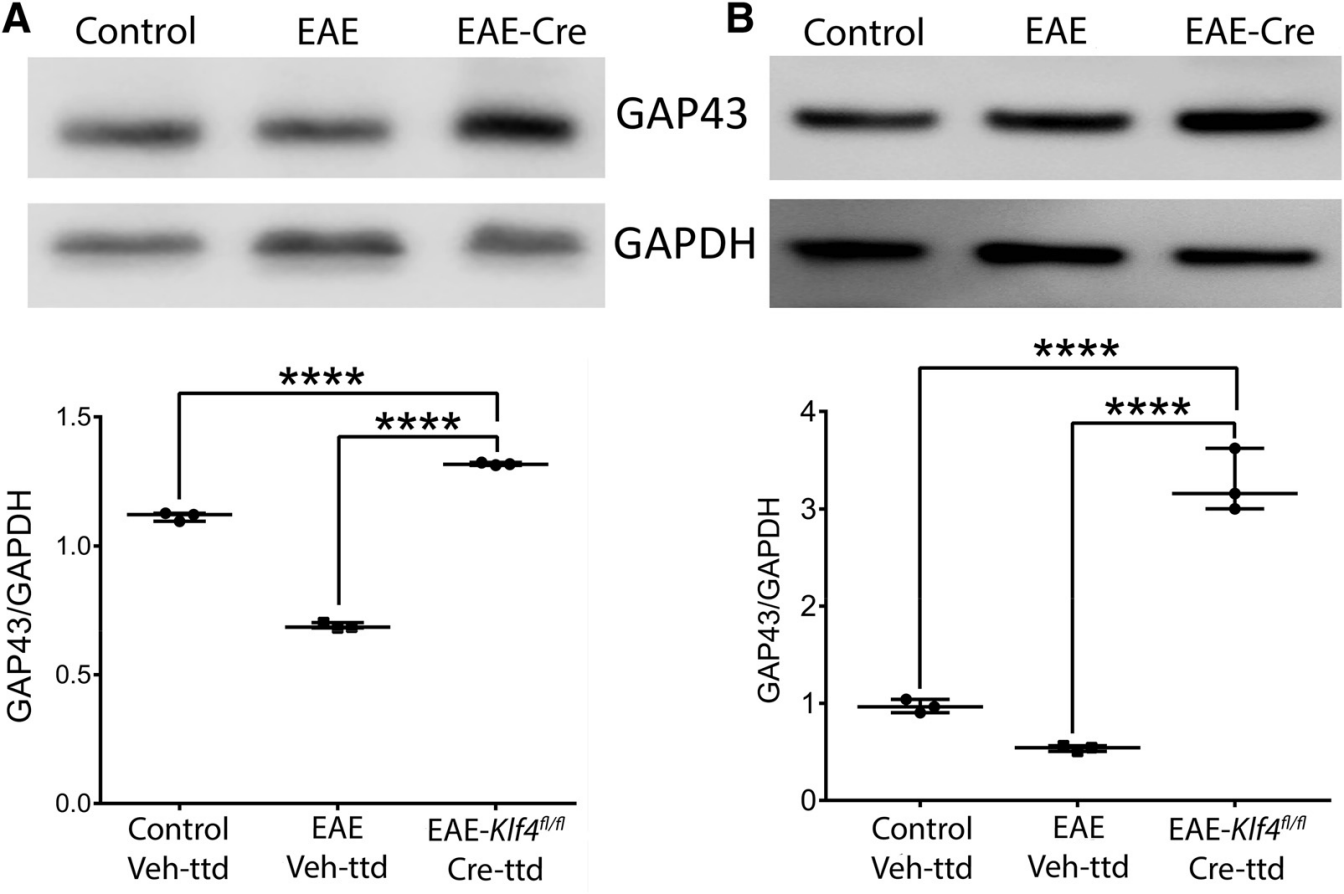
Axonal regeneration: immunoblotting of (***A***) ON and (***B***) retina samples obtained from 18 MPS/9 MPI of control-Veh-ttd, EAE-Veh-ttd, and EAE-*Klf4^fl/fl^*-Cre-ttd mice shows expression of GAP43 (top panels) and GAPDH (bottom panels) in all samples. GAP43 band intensities were higher in EAE-*Klf4^fl/fl^*-Cre-ttd mice compared with control or EAE Veh-ttd mice. Corresponding box-whisker plots at the bottom of the blots shows the ImageJ-based quantitative analysis of GAP43/GAPDH expression in ON and retina among three groups. Samples derived from the same experiment and that gels/blots were processed in parallel for quantitative analysis. Statistical analysis was performed by one-way ANOVA followed by Tukey’s multiple comparison test, a *p* < 0.05 is considered to be statistically significant; ****p < 0.0001–0.0009 (*n* = 6 eyes/group, experimental repeats = 3).*Figure Contributions*: Venu Talla acquired the data, analyzed, and generated the figure.

To gauge RGC axon regeneration and axon numbers, we used immunoblotting of GAP43 as a common surrogate marker for axonal regeneration, as well as transmission electron microscopic (TEM) analysis of ON samples obtained from the control-Veh-ttd, EAE-Veh-ttd, and EAE-*Klf4^fl/fl^*-Cre-ttd mice euthanized after 18 MPS/9 MPI. Immunoblotting detected GAP43 expression in both ON (Fig. 5*A*) and retina (Fig. 5*B*) samples of all three groups tested (Fig. 5). However, ImageJ-based quantitative analysis of the GAP43 band intensities relative to GAPDH showed a significantly higher GAP43 ratio in EAE-*Klf4^fl/fl^*-Cre-ttd mice compared with control and EAE littermates, indicative of active axonal regeneration in *Klf4*KO RGCs (*p* < 0.0001; Fig. 5).

**Figure 6.**
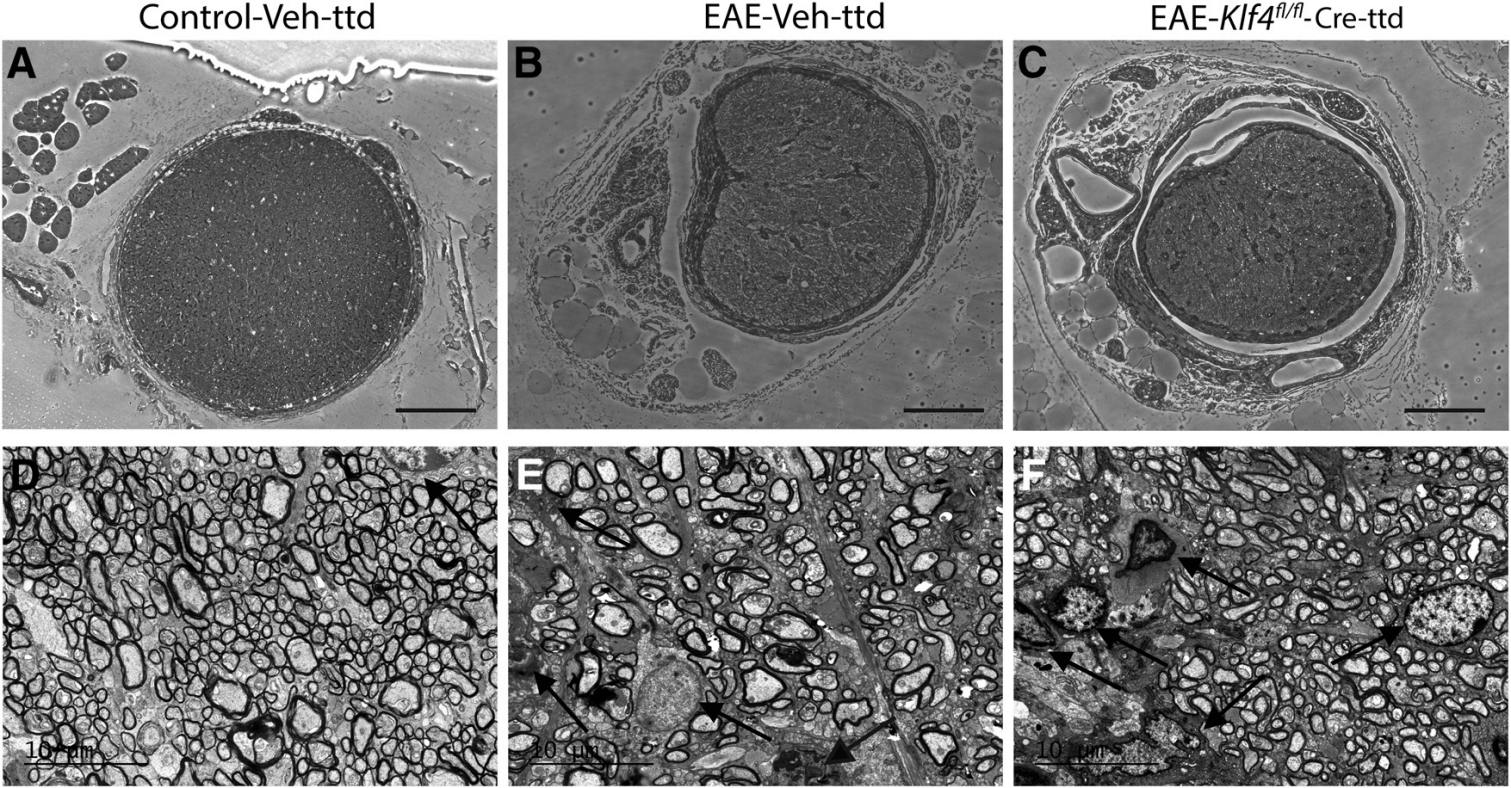
Histopathology and inflammation in EAE mice: ***A–C***, Light microscopic images of the toluidine blue-stained ON sections obtained 18 MPS/9 MPI show normal healthy axons in control-Veh-ttd mice (***A***), whereas inflammatory infiltration and fibrosis seen in EAE-Veh-ttd (***B***), and EAE-*Klf4^fl/fl^*-Cre-ttd mice. ***D–F***, Representative transmission electron micrographs show minimal to no cellular infiltration in control-Veh-ttd mice (***D***), whereas infiltration of astroglial cells was more evident in EAE-Veh-ttd (***E***) and EAE-*Klf4^fl/fl^*-Cre-ttd (***F***) mice ONs. Scale bars: 100 μm (light micrographs) and 10 μm (TEM images).*Figure Contributions*: Venu Talla and Rajeshwari Koilkonda acquired the images. Venu Talla generated the figure.

The ONs obtained from control-Veh-ttd, EAE-Veh-ttd, and EAE-*Klf4^fl/fl^*-Cre-ttd mice after 18 MPS were further used for histologic and TEM analysis. Light microscopic analysis of toluidine blue stained ON sections showed normal appearing axons and myelin in control-Veh-ttd mice (Fig. 6*A*) whereas, inflammatory cell infiltration and tissue fibrosis were evident in EAE-Veh-ttd (Fig. 6*B*) and EAE-*Klf4^fl/fl^*-Cre-ttd mice ONs (Fig. 6*C*). Further, transmission electron micrographs showed very minimal cellular infiltration in control-Veh-ttd ONs (Fig. 6*D*). In contrast, EAE characteristic astroglial cell infiltration and fibrosis were robust in EAE-Veh-ttd (Fig. 6*E*) and EAE-*Klf4^fl/fl^*-Cre-ttd mice ONs (Fig. 6*F*).

**Figure 7.**
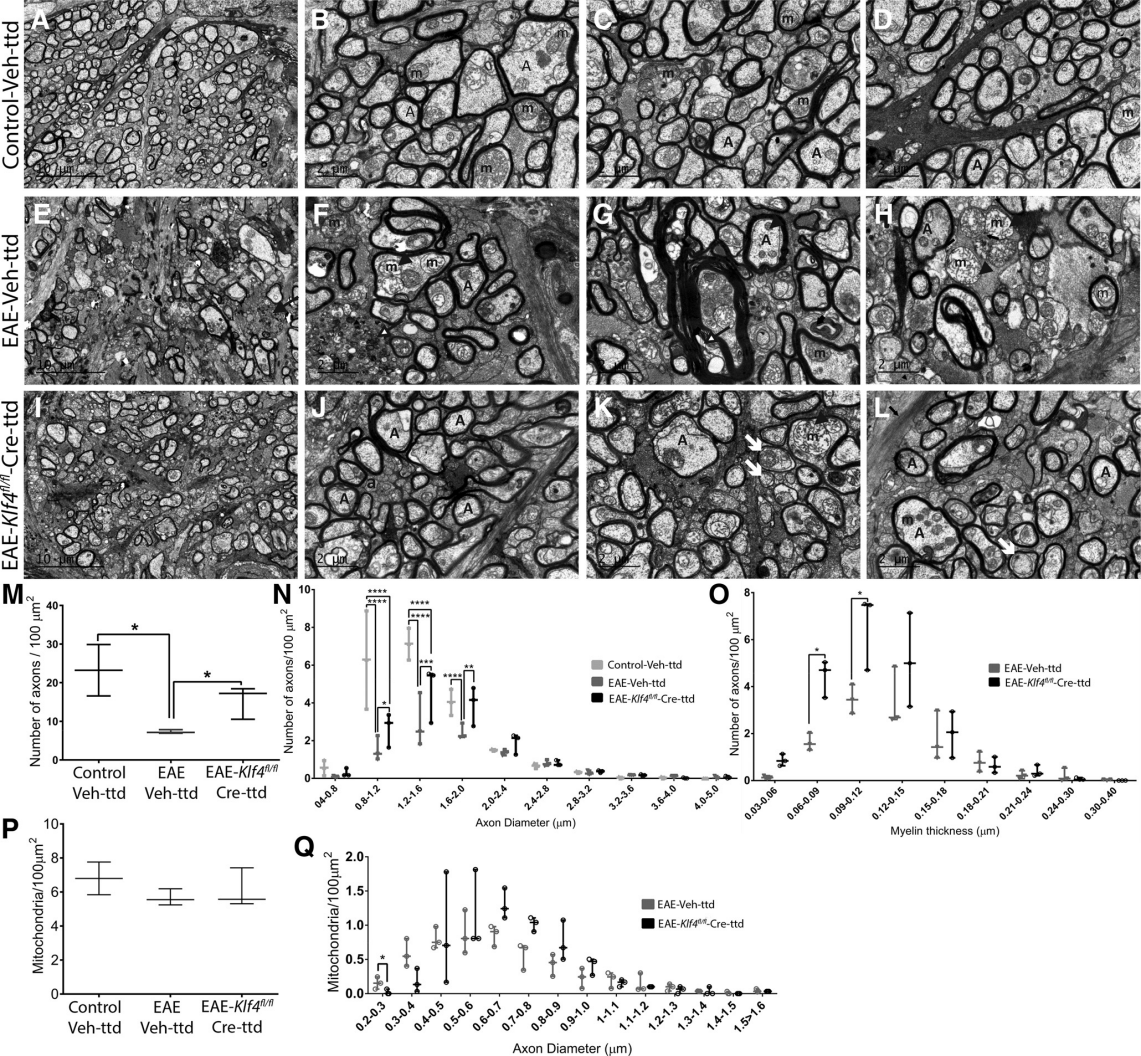
Axon counts, myelin, and mitochondria. Electron microscopy images of ON cross sections of (***A–D***) control-Veh-ttd mice shows healthy myelinated axons with normal mitochondria. ***E–H***, EAE-Veh-ttd mice showed axonal loss and Wallerian-like degeneration (as shown by arrows in ***E***, ***F***), myelin degradation (as shown in ***G***), and axons with either smaller or swollen mitochondria (shown with arrow heads in ***G***, ***H***). ***I–L***, EAE-*Klf4^fl/fl^*-Cre-ttd mice ON with low caliber axons, axons with thinner myelin (showed with arrows in ***K***), and normal as well as swollen mitochondria (arrowheads in ***K***) were evident in EAE-*Klf4^fl/fl^*-Cre-ttd mice. ***M***, Box and whisker plots compare axon counts among the three groups. ***N***, Box and whisker plots compare the distribution of small to high diameter axons per 100-μm^2^ area in control-Veh-ttd, EAE-Veh-ttd, and EAE-*Klf4^fl/fl^*-Cre-ttd mice. ***O***, Box and whisker plots compare the distribution of axons with low to high myelin thickness per 100-μm^2^ area in EAE-Veh-ttd versus EAE-*Klf4^fl/fl^*-Cre-ttd mice. ***P***, Box and whisker plot compares the number of mitochondria per 100-μm^2^ area in control-Veh-ttd, EAE-Veh-ttd, and EAE-*Klf4^fl/fl^*-Cre-ttd mice ONs. ***Q***, Box and whisker plot compares the mitochondrial number per 100-μm^2^ area with smaller to higher size in EAE-Veh-ttd versus EAE-*Klf4^fl/fl^*-Cre-ttd mice ONs. TEM imaging and axon counting was done by individual masked for the treatment groups. Statistical analyses were performed by one-way ANOVA followed by Tukey’s multiple comparison test for axon counts and mitochondrial counts. Two-way ANOVA followed by Tukey’s multiple comparison test was done for axon diameters. Two-way ANOVA followed by *t* test and statistical significance determined using the Holm–Sidak method, with α = 5% and computations assume that all rows are sample from populations with the same scatter (SD) was used for myelin thickness and mitochondrial size distribution analysis; *p* < 0.05 is considered to be statistically significant (*n* = 3 eyes/group, *p = 0.01–0.05, **p = 0.001–0.009, ***p = 0.0001–0.0009, ****p < 0.0001). A, axons; m, mitochondria. Scale bars: 10 μm (low-magnification images) and 2 μm (high-magnification images).*Figure Contributions*: Venu Talla and Rajeshwari Koilkonda acquired the images. Rajeshwari Koilkonda did the counting and diameters and myelin thickness measurements. Venu Talla and Rajeshwari Koilkonda analyzed the data. Venu Talla generated the figure.

TEM images revealed normal myelinated axons in ONs of control-Veh-ttd mice (Fig. 7*A–D*), whereas all the hallmark features of MS, including the axonal loss, Wallerian-like axonal degeneration, demyelination, myelin degradation, axons with multiple degenerating mitochondria or lysosomes, small and/or swollen mitochondria with disoriented cristae, were evident in ONs of EAE-Veh-ttd mice (Fig. 7*E–H*). Nevertheless, unmyelinated axons and axons with thinner myelin were observed in higher percentages in EAE-*Klf4^fl/fl^*-Cre-ttd mice (Fig. 7*I–L*). Quantitative analysis showed a 68.6% loss of axons in ONs of EAE-Veh-ttd (7.29 ± 0.27/100 μm^2^; average ± SE; *p* = 0.01; *n* = 3) and 33.6% loss in EAE-*Klf4^fl/fl^*-Cre-ttd mice (15.4 ± 2.45/100 μm^2^; *p* = 0.16; *n* = 3) compared with control-Veh-ttd control mice (23.2 ± 6.6/100 μm^2^; average ± SE; *n* = 3; Fig. 7*M*). The axonal numbers in EAE-*Klf4^fl/fl^*-Cre-ttd ONs were significantly higher than EAE-Veh-ttd mice, indicating possible axonal rescue or regeneration on targeted *Klf4* KO in RGCs of EAE-*Klf4^fl/fl^*-Cre-ttd mice (*n* = 3; *p* < 0.03; Fig. 7*M*). When axon diameters were characterized, axons with 0.8–2.0 μm in diameter (smaller diameter axons) were significantly lost in EAE-Veh-ttd and EAE-*Klf4^fl/fl^*-Cre-ttd mice compared with control-Veh-ttd (*p* < 0.0001). However, we noted a significantly higher number of small caliber axons (range of 0.8–2.0 μm) in EAE-*Klf4^fl/fl^*-Cre-ttd mice ONs (*p* < 0.05) compared with EAE-Veh-ttd, indicating possible axonal regeneration or rescue of these axons on KLF4-KO (Fig. 7*N*). Presence of unmyelinated axons and axons with thin myelin in the range of 0.06–0.12 μm were significantly higher in EAE-*Klf4^fl/fl^*-Cre-ttd mice compared with EAE-Veh-ttd mice (*p* < 0.05), further suggesting active axonal regeneration and remyelination in EAE-*Klf4^fl/fl^*-Cre-ttd mice (Fig. 7*O*).

Mitochondria numbers per 100-μm^2^ area of the ON showed no significant changes among the groups (Fig. 7*P*). However, small sized mitochondria (diameters <0.4 μm) were found significantly higher in EAE-Veh-ttd mice compared with EAE-*Klf4^fl/fl^*-Cre-ttd mice, *p* = 0.03, pointing toward a pathologic mitochondrial fission and degradation process in EAE mice ONs (Fig. 7*Q*). Normal sized mitochondria (0.5–1 μm in diameter) were relatively higher in EAE-*Klf4^fl/fl^*-Cre-ttd mice compared with EAE-Veh-ttd mice indicating possible role for KLF4 in mitochondrial homeostasis.

## Discussion

The axonal and neuronal degenerations responsible for progression of the disease begins early in optic neuritis/MS patients (Kuhlmann et al., 2002; Bjartmar et al., 2003). Despite anti-inflammatory therapies these patients progress to the irreversible permanent disability with no remedy (Noseworthy, 2003; Shirani et al., 2012). MS/EAE pathogenesis involves primary axonal demyelination and axon damage which, leads to the initial loss of function that subsequently contributes to neuronal degeneration and permanent disability. Since the PERG amplitude used in the current study can detect RGC functional decline well before neuronal loss due to EAE mediated injury, it allowed us to test our pro-regenerative approach of AAV-Cre-GFP-mediated KO of *Klf4* gene to rescue and induce axonal regeneration from non-functional injured RGCs, with the ultimate goal to revert the visual loss in these mice (Banitt et al., 2013). Our work here demonstrates that postinjury KO of *Klf4* gene in RGCs of EAE mice improved the visual function by increasing the ON axonal regeneration and/or rescue. Expression of KLF4 in CNS neurons including RGCs of the retina is developmentally regulated, beginning at postnatal day one and is found to be inhibitory to the axon growth and regeneration (Moore et al., 2009). Increased GAP43 expression and presence of greater number of small diameter axons observed in EAE-*Klf4^fl/fl^*-Cre-ttd mice ONs compared with Veh-ttd EAE mice in the current study, supports previous findings that the KO of *Klf4 in vitro* in adult primary CNS neurons and *in vivo* in ON crush models improves the axonal regeneration (Moore et al., 2009, 2011). Yet path-finding of regenerated axons in injured CNS poses a great challenge and various studies exploring the axon regenerative approaches in ON crush model showed regenerated axons going in different directions (Sun et al., 2011; Luo et al., 2013; Mehta et al., 2016), including some innervating the appropriate target, the lateral geniculate nuclei (Bei et al., 2016). Unlike in ON crush model, where the axonal injury is precisely marked and tracing the path of regenerated axon possible (Sun et al., 2011; Mehta et al., 2016), the axonal injury in EAE/MS is more diffuse and tracing the path of any regenerated axons is more difficult and can possibly be studied only by overall functional improvement. In the current study, we observed increased PERG amplitudes and preservation of RGCs on *Klf4*-KO in EAE mice retina that improved RGCs function which can be attributed to some of the regenerated RGC axons possibly reaching their targets.

KLF4 expression has multitude of effects on various neurologic and autoimmune diseases including EAE/MS. Depending on the type of cells and conditions under which it is expressed, it can regulate diverse cellular processes like proliferation, differentiation, development, maintenance of normal tissue homeostasis and apoptosis (McConnell and Yang, 2010; Moore et al., 2011; Ghaleb and Yang, 2017). KLF4 regulates the differentiation of interleukin (IL)-17 expressing CD4^+^ T cells that are known to play a critical role in MS/EAE pathogenesis. It controls the differentiation of Th17 cells by regulating the expression of IL-17 (An et al., 2011). Accordingly, T cell-specific *Klf4*-KO mice are resistant to induction of EAE due to impaired Th17 differentiation (An et al., 2011; Hao et al., 2017). KLF4 is also involved in pro-inflammatory activation of classically activated M1 macrophages, and regulates the polarization of pro-inflammatory M1 macrophages to anti-inflammatory M2 macrophages which are found to play major role in EAE/MS pathogenesis (Feinberg et al., 2005; Liao et al., 2011). Increased expression of KLF4 in T cells, macrophages, neurons, active astroglia of EAE rat spinal cord (Ahn et al., 2015) and other auto-immune disease models including Alzheimer’s (Li et al., 2017), traumatic brain injury (Cui et al., 2017) was associated with increased loss of neurons. In addition, increased expression of KLF4 under disease conditions involving ROS result in p53 mediated cell death (Cui et al., 2017; Li et al., 2017). In support of these observations, in the current study, we noted relatively higher number of live RGCs in *Klf4-KO* EAE mice compared with mock treated EAE mice, indicating deletion of KLF4 not only helps in axon regeneration but also appears to be protective to RGCs in EAE. However, the effect of KLF4 on neurite and axon regeneration was found to be independent of its cell survival functions (Moore et al., 2009, 2011). Klf4-KO in hippocampal neurons or embryonic RGCs or in RGCs of ON crush model showed no effect on the cell survival (Moore et al., 2009, 2011).

A new role for KLF4 in mitochondrial homeostasis and biogenesis is recently emerging (Jang and Arany, 2015). *Klf4*-KO in heart and kidney under various disease conditions is shown to be deleterious to mitochondrial function and homeostasis (Liao et al., 2015). In contrast, overexpression of KLF4 in embryonic and post-natal RGCs resulted in reduced mitochondrial DNA replication and mitochondrial bioenergetics (Steketee et al., 2012). In our study, we noticed higher amounts of normal sized mitochondria in *Klf4*-KO ON axons, whereas higher number of small sized and swollen mitochondria were found in KLF4 intact EAE ONs, indicating mitochondrial abnormality and dysfunction consistent with earlier studies. The KLF4 role in mitochondrial biogenesis and homeostasis appears to be tissue type and context dependent and incompletely understood and needs further investigation.

The limitations of the current study include the following. First, the mice were not scored on a traditional five-point EAE scale, as sensitization with homologous spinal cord emulsion in CFA result in a chronic model where the disease starts with milder symptoms and develop progressive axonal and neuronal degenerations. The EAE model using homologous spinal cord emulsion is well characterized and extensively published (Rao, 1981; Guy et al., 1992; Qi et al., 2006).Second, the KLF4 protein expression was evaluated at one month after Cre-GFP injection and we do not know when along the time course the KLF4 protein KO occurred and if it reached >70% at later time course. Incomplete KLF4 protein KO observed in Western blottings of Cre-GFP-injected mice (i.e., 70%) can presumably be either due to incomplete targeting of RGCs by AAV2-Cre-GFP virus or due to the contribution of KLF4 from other cell types in the retina. We did not use the contralateral eye as control, mainly to avoid the possible interference while recording PERG for RGC functional assessment that could potentially mislead the data.

In the current study, we primarily focused on ON, mainly because the ON lesion in EAE mice directly correlates to the loss of function and can be monitored in live mice using noninvasive techniques like PERG and SD-OCT (Sergott, 2005; Trip et al., 2005). *Klf4*-KO can be easily achieved in RGCs and ON of double floxed *Klf4* mice by intravitreal injection of AAV2 Cre recombinase virus (Zhong et al., 2008), which allows to study the effect of Klf4-KO specifically in RGC neurons. By changing AAV serotype for example to AAV9 which targets brain and spinal cord, the entire CNS can be targeted in EAE/MS for further studies (Duque et al., 2009). With the advent of efficient CRISPR mediated genome editing techniques for inactivation of target gene, it is now possible to undertake studies which could translate into the clinic (Xu et al., 2018). Taken together, our findings suggest that it is worth exploring the possible axonal regenerative approaches to counter permanent disability in MS/EAE.

## Synthesis

Reviewing Editor: Kirill Martemyanov, The Scripps Research Institute Decisions are customarily a result of the Reviewing Editor and the peer reviewers coming together and discussing their recommendations until a consensus is reached. When revisions are invited, a fact-based synthesis statement explaining their decision and outlining what is needed to prepare a revision will be listed below. The following reviewer(s) agreed to reveal their identity: Tudor Badea, Maureen McCall.

Both reviewers agree that the study reports potentially interesting findings related to mechanisms of retina ganglion cell survival. Several strengths of the study were noted and overall the reviewers’ were supportive. As noted below - reviewer #1 placed more emphasis on conceptual advances yet raised a number of significant points that need to be addressed. Reviewer#2 was distracted by a flaws related to presentation of the data - thus raising significant technical concerns. It was agreed that the manuscript needs to be thoroughly revised addressing all of the indicated issues and perhaps more importantly substantially revamping the presentation of the results so that the significance of the findings could be more fully appreciated. Please refer to unedited comments of made by the reviewers below.

In addition to these issues please re-evaluate your statistical approaches. Specifically, the reviewers mention the need to explicitly state whether all analyzed data sets were normally distributed by KS, which is a pre-requisite for using ANOVA. The box and whiskers distribution plots for many of the data call normality into question. Additionally, in several places where ANOVA is performed, there is another variable (time) and a two way ANOVA would be more appropriate, if the data are normally distributed.

Reviewer #1

The authors report the surprising and potentially therapeutically interesting finding that removing Klf4 specifically from retinal neurons has a protective effect on Retinal Ganglion Cell (RGC) survival and function in experimental autoimmune encephalomyelitis (EAE). EAE is experimentally induced by auto-immunization with homogenized nervous tissue rendered immunogenic using Freund’s adjuvant. It is characterized by inflammatory infiltrates in various central nervous system subdivisions, including the Optic Nerve (ON), which contains the axons of RGCs tracking from the eye to the brain, sheathed in myelin generated by Oligodendrocytes of the ON.

The authors provide evidence from Patterned Electroretinograms - thought to reveal RGC activity - that removing Klf4 from RGCs (or more likely from the whole retina) results in dramatic functional rescue of RGCs. In fact, data in Figure 2 suggests that retina specific Klf4-KO mice have even better PERGs compared to control animals, depending on the assayed ages.

The authors use Optical Coherence Tomography (OCT) to show that the thickness of the Ganglion Cell Layer (containing RGCs and Amacrine Cells) and the Inner Plexyform Layer (containing the dendrites of RGCs and Amacrine Cells, and the axons of bipolar cells) is reduced in EAE, but restored by deletion of Klf4. In conjunction with this, RGC numbers, expression of GAP43 and numbers of axons in Optic Nerve are dramatically reduced in EAE and restored by Klf4 ablation. Within the Optic Nerve, RGC axon diameter and mitochondria numbers and size are significantly affected by EAE, and restored by Klf4 ablation.

Overall, this seems to be a very well conducted study, with solid numbers of animals for each experimental condition, and a somewhat surprising conclusion. Mechanistically, EAE is characterized by sever inflammatory process of the CNS, and Klf4 has been previously implicated in the pathogenesis of the disease by modulating the responses of inflammatory cells, ranging from the Th-17 subset of CD4 T lymphocytes, to macrophages, astrocytes and other types of resident monocytes and glia. This study now claims that ablation of Klf4 specifically from RGCs is sufficient to protect them from destruction.

Some questions regarding the mechanism of the observed phenotype and specifics to the data.

1) The authors did not document what happened to the inflammatory aspect of the disease in their experimental model. Are there infiltrates, fibrosis ? Related to this, there is no insight as to whether Klf4 was deleted from astrocytes or macrophages in the retina or optic nerve ?

2) Figure 1 and throughout - it would be ideal if full images of the representative Western Blots would be included. For instance, the Klf4 band in panel E seems to have some spotty pattern.

3) Figure 1 B and C, it would be ideal to separate the red and green panels, in order to show the double staining of eGFP (infected cells) and Thy-1 (RGCs).

4) Figure 2 - surprisingly, the PERG amplitude in panel A is higher in Klf4 ablated animals at 4 and 9 months compared to controls without EAE (so essentially WT, normal animals). How do the authors account for this effect ?

5) Figure 3 - OCT EAE result in more severe phenotype in the Klf4KO/WT (heterozygote) mice compared to Klf4 animals, regardless of Cre treatment (so either ablated or not). How do the authors interpret this ?

6) Figure 4 - whole mount immunostaining for RGCs: EAE retinas look like they have lost a large fraction of their GCL layer (which is made up of 50 % Amacrine cells). In addition, in sections, the INL and ONL are also disrupted, compared to the control. The authors report RGC counts / mm2, but it is unclear whether the cell loss is not covering more than just RGCs. Could other cells of the retina be affected ?

7) Figure 5 - Western blot in panel B shows that there is a reduction in GAPDH, rather than an increase in GAP43. Was the same amount of protein loaded?

8) A comment on the statistics. Normality was checked using the Kolmogorov-Smirnoff test, and then ANOVA was used to compare the groups. However it is not explicitly stated whether all analyzed data sets were normally distributed by KS, which is a pre-requisite for using ANOVA.

Reviewer #2

The authors present results that suggest that a reduction in Klf4 in retinal ganglion cells alters degeneration and decline in visual function.

The presentation of these results is not straightforward and it is very difficult to parse out what the comparisons mean vs. the authors interpretation of what the comparisons mean. Part of this results from the poor definitions of the abbreviations used for the treatment groups. Part of this results from the illogical presentation of the groups on the graphs. When one group is being compared to another those data should be grouped together. Some of the statistics are likely not appropriate and the authors should reexamine if there data sets are really normally distributed. If data from one group are not, a non parametric test should be applied. A two way ANOVA (or it non-parametric equivalent) should be applied when time is another variable.

They authors omit: 1. definitions throughout (EAE, MPI, MPS, CFA), 2. description of how the amplitude of the PERG was evaluated.

Expression: Figure 1 - Shows that there is a reduction in Klf4 (NOT an ablation). They should not refer to this condition as a KO. They do not know if all cells are reduced by 70% OR if 70% of the cells do not expression Klf4. This is important as the latter could be important to the interpretation of why they observe small axons in the Klf4 reduced retinas.

PERG data - Figure 2 - CFA at 9 mps is the same as CFA (18mps)-mCherry 9 MPI AND CFA (18mps)-mCherry 9 MPI is the same as EAE-9MPS BUT CFA at 9 mps IS DIFFERENT from EAE-9MPS. HOW CAN THIS OCCUR?

ARE these the same animals in the matched color groups? The important point here is whether the EAE Klf4(fl/fl) - 9 MPS and the same as in EAE-Klf4 (fl/fl)(18 MPS)-Cre GFP 9 MPI. If they are the n’s do not match. If they are not, why didn’t the authors use this experimental design?

OCT - Figure 3

It is unclear that the quality of their OCTs allows them to actually draw borders, and were the people doing that analyses blinded to the condition of the animal? why do the authors change colors of the conditions between figures?

Immuno - whole mounts and sections, why are the data in M and N plotted on different axes? What is control in N vs, CFA-Cherry in M? Could the authors make the labels on the axes within this figure consistent and while they are at it could they do the same across figures as well. Why do the authors switch to this type of plot when figures 2 and 3 have box and whisker plots? (same for Figure 5 & 6)

Figure 6 how did the authors draw the smoothed curves for the two data sets on each graph? Do the authors take the mean of the data from each optic nerve and then compute a grand mean over axons from different animals OR are they computing a mean from all of their measurements? If the latter, this inflates the n and increases the likelihood that small (and potentially biologically insignificant) differences become statistically significant. Without the box and whiskers plots, it is not possible to evaluate if the data are normally distributed OR how many data points are being plotted.

I cannot evaluate the discussion without a more clear idea of the results.
